# Quercetin and Kaempferol as Multi-Targeting Antidiabetic Agents against Mouse Model of Chemically Induced Type 2 Diabetes

**DOI:** 10.3390/ph17060757

**Published:** 2024-06-08

**Authors:** Muhammad Ali, Mudassir Hassan, Siddique Akber Ansari, Hamad M. Alkahtani, Lamees S. Al-Rasheed, Shoeb Anwar Ansari

**Affiliations:** 1 Department of Biochemistry, Faculty of Sciences, University of Agriculture Faisalabad (UAF), Faisalabad 38040, Pakistan; mudassirhassanshigri786@mail.com; 2 Department of Biotechnology, Akhuwat Faisalabad Institute of Research Science and Technology Faisalabad (A-FIRST), Faisalabad 38040, Pakistan; 3 Department of Pharmaceutical Chemistry, College of Pharmacy, King Saud University, P.O. Box 2457, Riyadh 11451, Saudi Arabia; sansari@ksu.edu.sa (S.A.A.); ahamad@ksu.edu.sa (H.M.A.); 442202895@student.ksu.edu.sa (L.S.A.-R.); 4 Department of Drug Science, Technology University of Turin, 10124 Turin, Italy; shoeb.ansari@edu.unito.it

**Keywords:** type 2 diabetes, molecular docking, anticancer activity, kaempferol, quercetin, multi-target compounds

## Abstract

Diabetes, a multifactorial metabolic disorder, demands the discovery of multi-targeting drugs with minimal side effects. This study investigated the multi-targeting antidiabetic potential of quercetin and kaempferol. The druggability and binding affinities of both compounds towards multiple antidiabetic targets were explored using pharmacokinetic and docking software (AutoDock Vina 1.1.2). Our findings showed that quercetin and kaempferol obey Lipinski’s rule of five and exhibit desirable ADMET (absorption, distribution, metabolism excretion, and toxicity) profiles. Both compounds showed higher binding affinities towards C-reactive protein (CRP), interleukin-1 (IL-1), dipeptidyl peptidase-4 (DPP-IV), peroxisome proliferator-activated receptor gamma (PPARG), protein tyrosine phosphatase (PTP), and sodium–glucose co-transporter-1 (SGLT-1) compared to metformin (the positive control). Both quercetin and kaempferol inhibited α-amylase activity (in vitro) up to 20.30 ± 0.49 and 37.43 ± 0.42%, respectively. Their oral supplementation significantly reduced blood glucose levels (*p* < 0.001), improved lipid profile (*p* < 0.001), and enhanced total antioxidant status (*p* < 0.01) in streptozotocin–nicotinamide (STZ-NA)-induced diabetic mice. Additionally, both compounds significantly inhibited the proliferation of Huh-7 and HepG2 (cancer cells) (*p* < 0.0001) with no effect on the viability of Vero cell line (non-cancer). In conclusion, quercetin and kaempferol demonstrated higher binding affinities towards multiple targets than metformin. In vitro and in vivo antidiabetic potential along with the anticancer activities of both compounds suggest promise for further development in diabetes management. The combination of both drugs did not show a synergistic effect, possibly due to their same target on the receptors.

## 1. Introduction

Diabetes mellitus (DM) is a complex metabolic non-communicable disorder arising from defective insulin secretion (type 1 DM), action (type 2 DM), or both, leading to an abnormal increase in blood glucose level [1]. The global prevalence of diabetes has reached epidemic levels. Currently, 415 million individuals (about 9% of the world’s population) are affected by diabetes globally, and is projected to cross 642 million by 2040 [2]. Reactive oxygen species (ROS) and disruption in antioxidant defense mechanisms have been implicated in the surge of diabetic cases [3]. Body mass index strongly correlates with the development of type 2 diabetes [4]. Abnormal glucose homeostasis and lipid metabolism are commonly observed in diabetic patients [5]. Chronic hyperglycemia often leads to serious complications affecting vital organs like eyes, liver, kidney, and heart [6]. Type 2 diabetes, often referred to as non-insulin-dependent diabetes mellitus (NIDDM), is directly linked with the development of various types of cancer in the pancreas, liver, and breast [7].

Implementing lifestyle adjustments and weight maintenance supplemented with pharmacological interventions are required in type 2 diabetes management. These interventions include stimulators for insulin release (e.g., sulfonylureas), insulin analogs, insulin sensitizers (e.g., thiazolidinediones), and inhibitors of liver glucose absorption and production (e.g., acarbose and biguanides) [8]. Metformin remains the primary therapy, although other options like sodium–glucose co-transporter (SGLT) inhibitors, gliptins, glitazones, and glucagon-like peptide 1 (GLP-1) agonists are also commonly prescribed [9,10]. However, the increasing diabetic population, high medical cost, and potential adverse effects of pharmaceutical drugs used for the management of diabetes pose considerable challenges [9,11].

With the increasing understanding of complex diseases, the process of drug discovery has shifted from the old established “one drug” for “one target” paradigm towards the emerging “multi-target approach” [12]. The conventional single-target therapeutic approach often falls short in tackling the intricate pathophysiology where multiple factors are involved in the progression of the disease [1,13]. Moreover, the therapeutic potentials of multi-targeting drugs can be higher and effective at lower doses in a synergic manner [14]. Therefore, the discovery of cost-effective multi-targeting drug candidates with safety profiles can be an effective option to combat multifactorial diseases like diabetes [13].

In drug discovery, both the computational and in vivo approaches are valuable and reinforce each other [15]. In silico computer-aided drug designs and bio-informatics tools have significantly reduced drug discovery time and cost [16]. In silico screening and optimization of drug candidates based on druggability and exclusion of chemicals/ligands having undesirable absorption, distribution, metabolism, excretion, and toxicity (ADMET) profiles are desired in drug discovery [15,17,18]. Molecular docking is commonly used in identifying potent drug candidates in drug discovery, enabling the atomic-level prediction of ligand–receptor interactions [19].

Plant-derived remedies have gained attention in recent years due to their antioxidant properties and lower toxicity [20,21]. Apart from orally taken antidiabetic drugs like metformin and sulfonylurea, different parts of the plant are also used to manage diabetes worldwide [22]. Metformin, often referred to as dimethylbiguanide, is commonly used as the first-line oral treatment for diabetes. Its roots can be traced back to *Galega officinalis*, a European herbal remedy discovered in 1918 for its ability to treat hyperglycemia [23]. Flavonoids, as major secondary metabolites in edible plants, have drawn significant interest for their health benefits in combating various ailments [24]. Due to their antioxidant, anti-inflammatory, and neuroprotective qualities, plants that contain polyphenols and polyflavonoids have shown encouraging results in treating various disorders [24].

This study investigated the antidiabetic potentials of quercetin and kaempferol (flavonoids) against multiple targets crucial in the progression of diabetes. Quercetin is commonly found in dietary sources such as fruits, vegetables, herbs, and cereal grains [25]. Its biological activities of quercetin include antioxidant, anticancer, antiviral, anti-inflammatory, anti-obesity, and anti-allergic properties [25,26,27,28,29,30]. Kaempferol, naturally found in barberries, citrus, apples, citrus fruits, strawberries, and onions, is known for its antioxidant, anticancer, antimicrobial, neuroprotective, and hepatoprotective effects [31,32,33].

## 2. Results

### 2.1. In Silico Studies

#### 2.1.1. Drug Scan and ADMET Profiling

**Figure 1:**
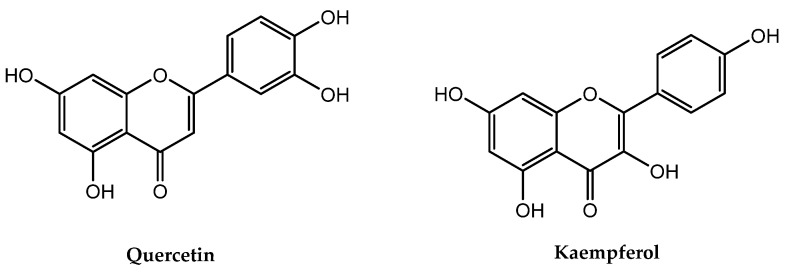
Chemical structure of quercetin and kaempferol.

The druggability of potential drug candidates can be evaluated using the Lipinski’s Rule of Five (Ro5) [34]. This rule, also known as Pfizer’s rule, sets certain criteria such as that the molecular weight (Mol. WT) should be less than 500 Daltons, the number of hydrogen bond donors (HBDs) should be no more than 5, the number of hydrogen bond acceptors (HBAs) should be less than 10, the number of rotatable bonds (RBNs) should be 10 or less, the octanal water partition coefficient (LogP) should no more than 5, and the molar refractivity (A) should be between 40 and 130. This rule evaluates the drug-likeness of a compound, indicating its potential pharmacological effectiveness and oral activity in the human body based on its pharmacokinetics and physicochemical parameters. Usually, compounds with no violation or minimal violation (not more than one) ensure potential activity, while two or more violations suggest limited oral effectiveness for drug candidates [17]. Both quercetin and kaempferol in our study followed Lipinski’s Ro5, showing no violation as shown in Table 1. Metformin, the widely used antidiabetic drug, served as a reference. The chemical structures of both compounds are shown in Figure 1.

Further, ADMET analysis of quercetin, kaempferol, and metformin (standard drug) was performed. ADMET profiling is important in predicting the absorption, distribution, metabolism, excretion, and toxicity of candidate drug molecules in the field of medicinal chemistry, providing valuable insights for drug development [35,36]. Drug candidates with unfavorable ADMET profiles are excluded from clinical trials in the process of drug discovery [18]. Both quercetin and kaempferol exhibited consistent results regarding the blood–brain barrier (BBB) and absorption in the human body. Additional models such as human intestine absorption (HIA), renal organic cation transporter, and P-glycoprotein substrate evaluations also aided in assessing the potential of these compounds as effective drug candidates. Another important model involves clusters of the cytochrome P50 isoenzyme, which plays a significant role in drug metabolism, with approximately 75% of drug metabolism associated with these enzyme clusters, showing a suitability of quercetin and kaempferol. It was observed that both compounds were non-inhibitors of CYPEC9 and CYP2D6 enzymes of this enzyme family. Additionally, we observed a lower likelihood of the existence of P-glycoprotein substrate and inhibitor for all four compounds. Regarding the renal organic cation transporter (ROCT) substrate, potential advantages like reduced efflux, renal elimination, and improved bio-availability contributing to the desirable pharmacokinetic profile were also predicted. Further, admetSAR and Swiss ADME analysis indicated both compounds to be non-toxic and non-carcinogenic. The ADMET-related parameters quercetin, kaempferol, and metformin (standard antidiabetic drug) are compiled in Table 2.

#### 2.1.2. Molecular Docking

Molecular docking was carried out to explore the binding affinities of both compounds towards multiple antidiabetic targets using AutoDock Vina 1.1.2 software [37]. These targets included CRP, IL-1, DPP-IV, PPRG, PTP, and SGLT-1 [38,39,40,41,42,43,44]. Modulation of these targets is crucial in regulating blood glucose levels, insulin signaling, lipid metabolism, and inflammation in diabetes. CRP and IL-1 contribute to inflammation and inhibit insulin-mediated glucose transport. Inhibition of DPP-IV restores the function of glucagon-like peptide 1 (GLP-1) and glucose-dependent insulinotropic polypeptide (GIP) hormones crucial for glucose homeostasis, while PPARG activation improves insulin sensitivity and lipid metabolism. As shown in Table 3, quercetin and kaempferol had higher binding affinities towards all the targets than metformin. The binding affinities of quercetin and kaempferol ranged from ΔG = −5.8 to –8.4 (Kcal/mol) to ΔG = −4.2 to –8.4 (Kcal/mol), respectively. In contrast, the binding affinity of metformin (positive control) ranged between ΔG = −4.2 and –5.3 (Kcal/mol). Additionally, we observed no significant difference in the binding affinities of both quercetin and kaempferol against each target (Table 3).

**Figure 2:**
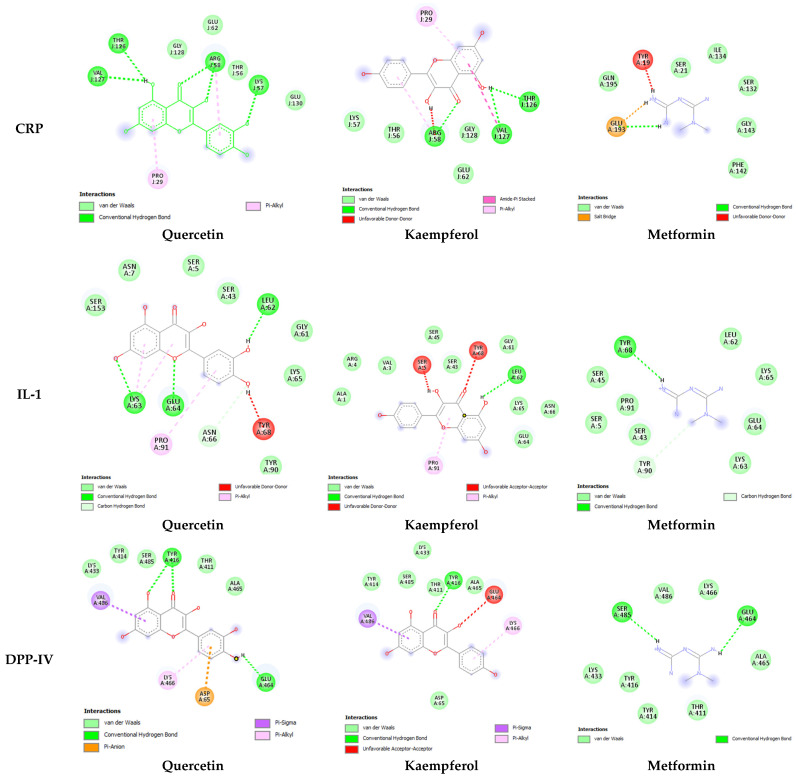
Two-dimensional representation of the interaction between quercetin, kaempferol, and metformin within the binding sites of CRP, IL-1, and the active site of DPP-IV. CRP (PDB ID: 1gnh), DPP-IV (PDB ID: 1j2e), and IL1 (PDB ID: 9ilb). Interaction analysis was carried out using BIOVIA Discovery Studio 2021 and the best pose was selected for each ligand.

**Figure 3:**
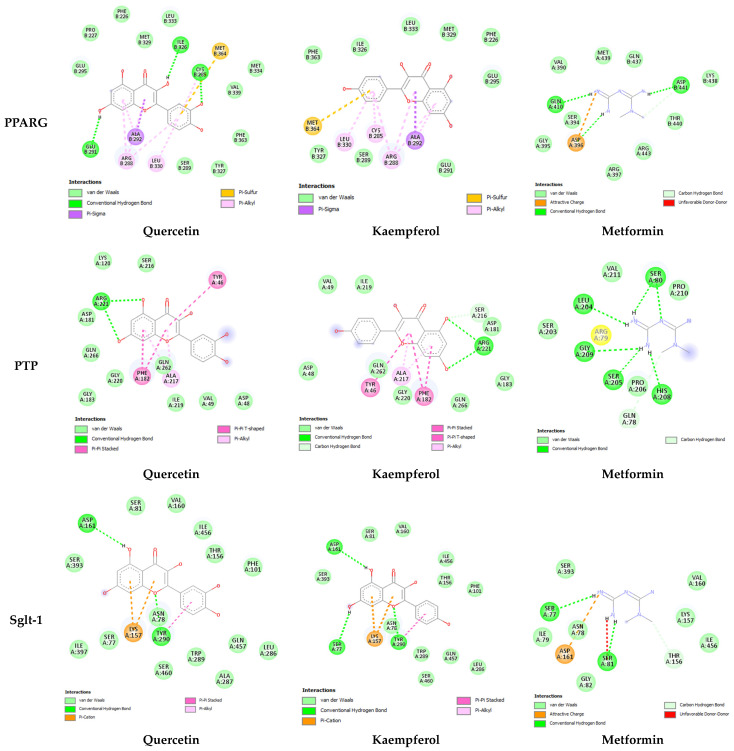
Two-dimensional representation of quercetin, kaempferol, and metformin interaction within canonical thiazolidinedione (TZD) binding sites of PPARG, active site of PTP, and binding site of SGLT-1. Quercetin (PubChem CID: 5280343), Kaempferol (PubChem CID: 5280863), and Metformin (PubChem CID: 4091). PRARG (PDB ID: 1prg), PTP (PDB ID: 2nt7), and SLGT-1 (PDB ID: 7sla). Interaction analysis was carried out using BIOVIA Discovery Studio 2021 and the best pose was selected for each ligand.

In-depth analysis of ligand–receptor interaction is important in the structure optimization of candidate drugs [15]. A careful examination of the ligand–receptor complex revealed diverse interactions for quercetin, kaempferol, and metformin, including conventional hydrogen bonds, van der Waals interaction, pi-alkyl, pi-sigma, amide pi-stacked, and hydrophobic interactions. The two-dimensional interaction between both these compounds as well as the standard drug with each antidiabetic target is presented in Figure 2 and Figure 3. The highest number of hydrogen bonds for quercetin was observed with CRP (5 H-bonds) (Figure 2). In comparison, kaempferol showed 3 H-bonds with CRP and SGLT-1.

### 2.2. In Vitro Anti-Hyperglycemic Activity Using α-Amylase Inhibition Assay

The inhibition of pancreatic alpha-amylase enzymes is one of the main targets in reducing the post-prandial increase in glucose levels [45]. This enzyme is responsible for breaking complex carbohydrates into simple sugars during digestion. Inhibition of this enzyme reduces the release of glucose in the bloodstream, especially after a meal. Both quercetin and kaempferol inhibited alpha-amylase up to 37.43 ± 0.42 and 20.30 ± 0.49%, respectively.

### 2.3. In Vivo Antidiabetic Activities

Motivated by the outcomes of in silico investigations, quercetin and kaempferol were further assessed for their in vivo antidiabetic activity using a streptozotocin–nicotinamide (STZ-NA)-induced diabetic mice model. Our in vivo study evaluated the effect of their oral supplementation on the experimental animal’s blood glucose levels, serum lipid profile, and hepatic antioxidant status.

#### 2.3.1. Effect of Quercetin, Kaempferol, and Their Combination on Blood Glucose Levels of Diabetic Mice

**Figure 4:**
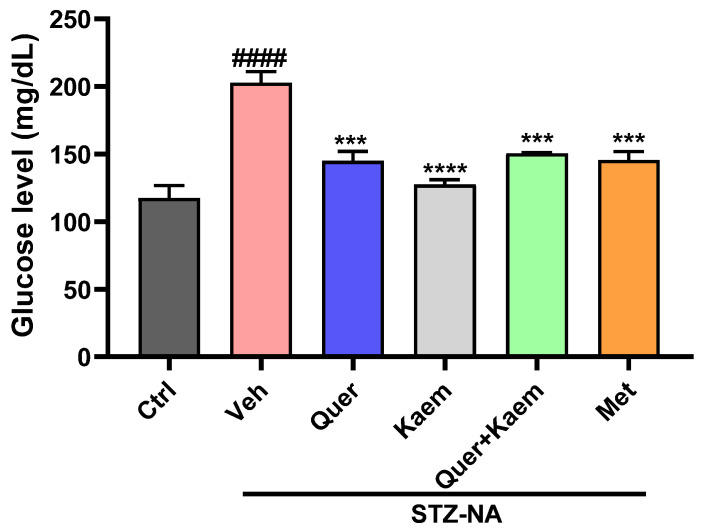
Graph showing the effect of quercetin, kaempferol, their combination, and metformin on blood glucose level. Data are expressed as mean ± SEM, where *n* = 3 (number of mice in each group). One-way ANOVA (analysis of variance) and Tukey’s multiple comparison test was applied to find the significance level and represented as *p*-value. *^####^ p* < 0.0001 shows comparison of diabetic control with normal control, *** *p* < 0.001 and **** *p* < 0.0001 show the comparison of other groups with diabetic control. Ctrl received 20% ethanol, representing normal control; Veh (vehicle) received normal saline, served as diabetic control; Quer received quercetin 20 mg/Kg; Kaem received kaempferol 5 mg/Kg; Quer + Kaem group received 10 mg/Kg of quercetin and 2.5 mg/Kg of kaempferol; and Met received metformin 50 mg/Kg served as positive control.

Blood glucose level serves as an ideal biomarker for monitoring and tracking the diabetic status of patients, as it fulfills the necessary criteria for this purpose [46]. The blood glucose level in all mice was increased compared to the control group after STZ-NA injection, confirming the successful induction of diabetes. Blood glucose level was significantly elevated (*p* < 0.001) in the STZ-NA-treated (diabetic control) group compared to the normal control (Ctrl) group. Oral administration of quercetin (20 mg/Kg), kaempferol (5 mg/Kg), and their combination significantly (*p* < 0.001) reduced blood glucose levels as compared to metformin (50 mg/Kg), as shown in Figure 4. The combination group was given 10 mg/Kg quercetin and 2.5 mg/Kg of kaempferol.

#### 2.3.2. Effect of Quercetin, Kaempferol, and Their Combination on Lipid Profile of Diabetic Mice

**Figure 5:**
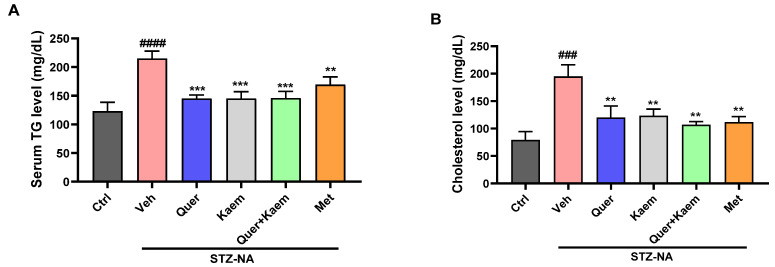
Effect of quercetin, kaempferol, and their combination on lipid profile; (**A**) serum triglyceride level and (**B**) cholesterol level of experimental mice. Data are presented as mean ± SEM, where *n* = 3. One-way ANOVA (analysis of variance) and Tukey’s multiple comparison test were applied to find the significance level and represented as *p*-value. *^####^ p* < 0.0001 and *^###^ p* < 0.001 show comparison of diabetic control with normal control, *** *p* < 0.001 and ** *p* < 0.01 show the comparison of other groups with diabetic control.

Dyslipidemia is often observed in diabetic patients and is associated with an increase in cardiovascular diseases [47]. The induction of diabetes caused a significant increase in serum triglyceride (TG) and total cholesterol (TC) levels in diabetic mice, which are key factors for the development of cardiovascular diseases. However, in the current study, quercetin, kaempferol, and their combination significantly regulated both serum TG and TC levels, respectively, as shown in Figure 5.

#### 2.3.3. Effect of Quercetin, Kaempferol, and Their Combination on Hepatic Markers of Diabetic Mice

The elevated levels of alkaline phosphatase (ALT), alanine aminotransferase (ALP), and bilirubin are associated with liver cell damage, leading to increased permeability and bringing these enzymes into the blood [48]. However, in this study, the induction of diabetes and the supplement of quercetin, kaempferol, and their combination showed no significant change in the serum ALT, ALP, and bilirubin levels, as shown in Table 4.

#### 2.3.4. Effect of Quercetin, Kaempferol, and Their Combination on Total Antioxidant Status (TAC) of Hepatic Tissues

**Figure 6:**
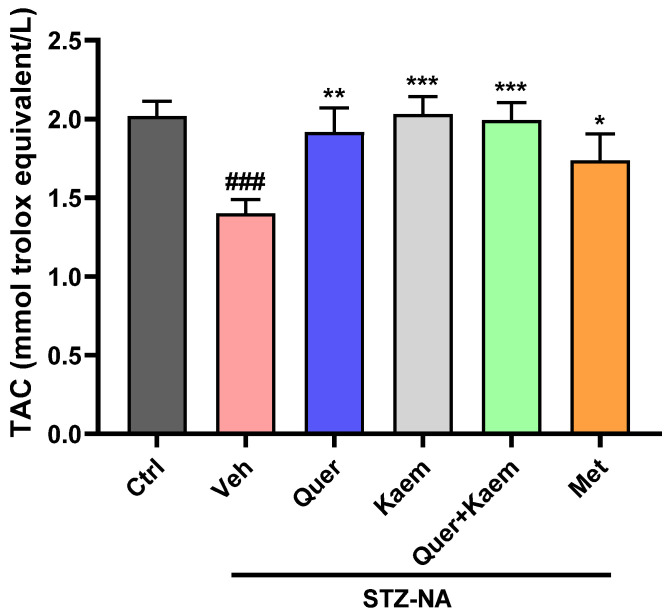
Effect of quercetin, kaempferol, and their combination on TAC status of liver tissue; values are means (*n* = 3) ± SEM, where *n* = 3. One-way ANOVA (analysis of variance) and Tukey’s multiple comparison test were applied to find the significance level and represented as *p*-value. *^###^ p* < 0.001 shows comparison of diabetic control with normal control, *** *p* < 0.001, ** *p* < 0.01, and * *p* < 0.05 show the comparison of other groups with diabetic control. Ctrl, normal control; Veh, vehicle (diabetic control); Quer, quercetin; Kaem, kaempferol; and Met, metformin (positive control).

Reactive oxygen species cause the oxidation of lipids as well as disturbance, leading to decreased total antioxidant status in diabetic conditions [49]. Depletion in antioxidant status of liver tissue was observed in diabetic control, which was significantly (*p* < 0.05) improved in quercetin, kaempferol, and their combination-treated mice. Interestingly, quercetin, kaempferol, and their combination all showed an improved total antioxidant status compared to metformin, as shown in Figure 6.

### 2.4. Cell Viability Assay

**Figure 7:**
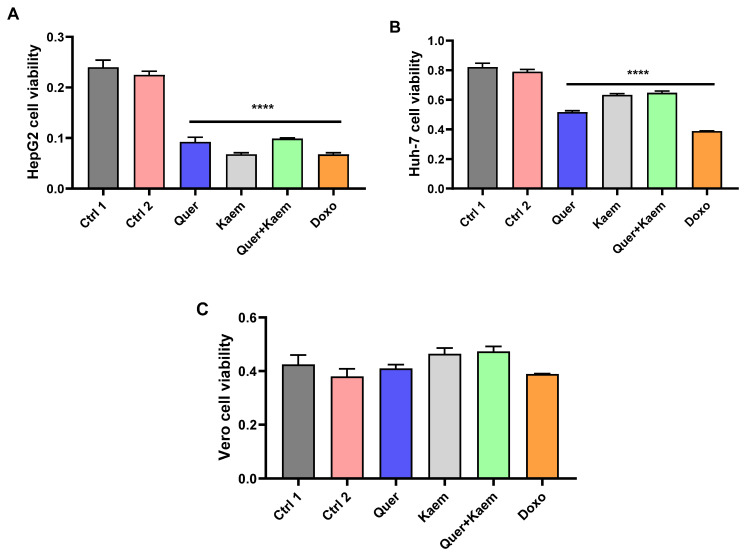
Effect of quercetin, kaempferol, and their combination on the viability of cancer and non-cancer cell lines. (**A**–**C**) Shows the effect of quercetin (36 µg/mL), kaempferol (15 µg/mL), their combination (18 µg/mL quercetin + 7.5 µg/mL kaempferol), and doxorubicin (1.8 µg/mL) on the viability of HepG2, Huh-7 (cancer), and Vero (non-cancer) cell lines, respectively. One-way ANOVA (analysis of variance) and Tukey’s multiple comparison test were applied to find the significance level and represented as *p*-value. **** *p* < 0.0001 shows comparison of all treatment groups with both controls. Ctrl1, control with distilled water; Ctrl2, control with 0.1–0.2% dimethyl sulfoxide (DMSO); Quer, quercetin; Kaem, kaempferol; and Doxo, doxorubicin as standard anticancer drug. The absorbance was measured at 570 nm.

Hyperinsulinemia is directly linked with the development of various types of cancer [50]. In this study, the cytotoxic effect of quercetin (36 µg/mL), kaempferol (15 µg/mL), and their combination (quercetin 18 µg/mL + 7.5 µg/mL) was explored against both cancer and non-cancer cell lines using an MTT assay. Quercetin, kaempferol, and their combination all significantly (*p* < 0.0001) inhibited the growth of Huh-7 and HepG2 cancer cell lines. However, no effect was observed on the viability of the Vero cell line (non-cancer). The cytotoxic activity of kaempferol was comparatively higher than quercetin in HepG2. It is evident from the MTT assay that the anticancer activity of both candidate drugs was comparable to doxorubicin (1.8 µg/mL), the standard anticancer drug (positive control), as shown in Figure 7.

## 3. Discussion

Diabetes comprises a complex pathophysiology involving various factors in progression [1]. The global diabetes epidemics and associated concerns about available medications demand the discovery of safer and more effective alternatives [9,11]. Multi-target drugs hold promise in addressing intricate conditions such as diabetes, captivating the interest of researchers. Since 2015, the FDA has increasingly approved such drugs, indicating their growing importance in pharmaceutical research [51]. Bioactive compounds derived from medicinal plants offer a promising option for the management of different ailments with fewer side effects. In the current research work, in silico molecular docking was carried out to explore the potential of quercetin and kaempferol as ligands against multiple antidiabetic targets including CRP, IL-1, DPP-IV, PPARG, PTP, and SGLT-1. These compounds were further tested for their antidiabetic activity using in vitro alpha-amylase inhibition assay and on blood glucose level, lipid profile, and total antioxidant status in STZ-NA-induced mice model of type 2 diabetes. Further, the cytotoxic activities of both compounds against both cancer and non-cancer cell lines were carried out using MTT assay.

The druggability and ADMET profiles of these compounds were explored in silico. The drug candidates with unfavorable ADMET profiles are excluded from clinical trials in drug discovery [18]. Both quercetin and kaempferol showed their drug-likeness as per Lipinski’s Rule of Five (Table 1) and displayed favorable ADMET profiles, showing no potential toxic or carcinogenic effect (Table 2). ADMET profiles of these compounds closely align with metformin. Their HIA, bio-availability, and ROCT substrate status suggested advantages such as reduced efflux, renal elimination, and improved bio-availability, which contribute to a desirable pharmacokinetic profile. An additional model of P-glycoprotein substrate, the P50 isoenzyme model involving clusters of the cytochrome associated with approximately 75% of drug metabolism, showed the suitability of both compounds as drug candidates. Moreover, both compounds were non-inhibitors of CYPEC9 and CYP2D6 enzymes of this family. Given these findings, using these bioactive compounds in drug formulation that possess druggable characteristics and a desirable ADMET profile can be more effective than synthetic compounds like acarbose. Acarbose has been reported to be metabolically unstable [52].

The molecular docking results further provided insight into the interaction modes and binding residues of both compounds at binding sites of CRP, IL-1, DPP-IV, PPARG, PTP, and SGLT-1 (Figure 2 and Figure 3). These targets are important in the regulation of hyperglycemia and related complications. CRP levels, often increased in diabetic individuals, play a pro-inflammatory role and suppress insulin-mediated GLUT4 (glucose transporter type 4) translocation. [39]. Interleukin (IL-I) is a member of the cytokine family associated with inflammation [53]. Inflammatory markers like IL-6, IL-1, tumor necrosis factor alpha (TNF-α), and CRP increase nuclear factor kappa b (NF-kb) and nitric oxide levels, thereby activating serine/threonine kinase and eventually causing impaired insulin signaling and loss of pancreatic β-cells [38,39]. An enhanced level of DPP-IV mostly observed in diabetes causes the cleavage of GIP and GLP-1, leaving them non-functional. Both GLP-1 and GIP are secreted in high glucose and play a vital role in glucose homeostasis by increasing insulin secretion from pancreatic β-cells [40]. DPP-IV inhibitors such as vildagliptin, sitagliptin, and saxagliptin are commonly used to treat type 2 diabetes. These inhibitors enhance the survival of GLP [54]. PTP overexpression in various organs, including skeletal muscles in type 2 diabetes, acts as a negative regulator of leptin and insulin signaling pathways [41]. PTP is reported to be involved in the de-phosphorylation of activated insulin receptors (IRs). The de-phosphorylation of IRS-1 and IRS-2 subsequently down-regulate the PI3K/AKT/GLUT4 signaling pathway [42]. SGLT-1 overexpression in diabetic conditions is responsible for intestinal and renal glucose absorption [43]. PPARG, also known as the glitazone receptor, is an important player in increasing fatty acid storage in fat cells, improving insulin sensitivity, and decreasing lipo-toxicity [44]. It is reported that PPARG agonists counteract the effect of TNF-alpha, which improves insulin resistance and enhances the transcription of genes involved in glucose and lipid metabolism [55]. The interaction analysis of both compounds with the selected targets showed conventional hydrogen bonds, van der Waals interaction, pi-alky, and pi-sigma interactions involved in ligand–receptor complex formation. Apart from hydrogen bonding and van der Waals interaction, hydrophobic interactions also appeared to promote and stabilize ligand–receptor interaction [52]. Both polar (Thr, Arg, Lys, Glu, Gln, Ser, Asn) and non-polar (Val, Gly, Leu, Ala) amino acids were involved in ligand–receptor complex formation (Figure 2 and Figure 3). It was worth noticing that the binding affinities of both quercetin and kaempferol were significantly higher than standard drug metformin (FDA-approved oral antidiabetic drug) (Table 3). Their higher binding affinities can be associated with the higher number of interacting amino acids at binding sites of the target. Additionally, both these compounds mostly interacted via the same residues at their targets. This suggests that particular amino acids are crucial for ligand–receptor complex formation. Moreover, the shared binding residues suggest similar binding modes and the same mechanism of action, indicating therapeutic effects through similar pathways.

In-depth analysis of their binding mode and residues at the binding sites/active sites of CRP, IL-1, DPP-IV, PTP, and SGLT-1 suggest that quercetin and kaempferol are in inhibition mode (Figure 2 and Figure 3). However, further investigation is required to confirm the presence of a full inhibitory activity for these compounds. Both compounds interacting with three reported residues (Cys285, Arg288, Ser289) at the canonical thiazolidinedione (TZDs) binding sites of PPARG (Figure 3) suggest their partial agonistic behavior. Candidates forming hydrophobic interaction with these residues of PPARG are reported to cause transactivation by stabilizing the H3 and β-sheet region, influencing co-activator recruitment [11,56].

Alpha-amylase enzyme converts carbohydrates into glucose, thereby increasing blood glucose levels. Inhibition of this enzyme is an important target in maintaining post-prandial control of hyperglycemia [45]. In this study, both quercetin and kaempferol inhibited alpha-amylase enzyme up to 20.30 ± 0.49 and 37.43 ± 0.42%, respectively. However, their percentage inhibition was lower than that of acarbose (positive control). The findings of Yang et al. (2023) support these findings, where a lower α-amylase inhibition activity of quercetin and kaempferol relative to acarbose is reported [57]. Further, the in vivo antidiabetic potentials of these compounds were assessed using the STZ-NA-induced mice model for type 2 diabetes. The blood glucose levels in all mice increased compared to the control group after the STZ-NA injection, confirming the successful induction of diabetes. The quercetin-, kaempferol-, and their combination-administered groups (orally) all showed significantly (*p* < 0.001) lower blood glucose levels (Figure 4), improved lipid profiles (Figure 5), and improved total antioxidant status (Figure 6) of diabetic mice when compared to the diabetic control group. Increased serum cholesterol and triglycerides were observed in STZ-NA-treated groups, which are key factors in the development of cardiovascular diseases [58,59]. The maintenance of total antioxidant status of a diabetic individual is essential in controlling complications associated with diabetes [60]. Decreased antioxidant status in diabetic individuals is reported to cause insulin resistance and lipid peroxidation, leading to damage to the pancreas and other vital organs [61]. The overall improvement in blood glucose, lipid profile, and antioxidant status observed in the experimental animals could have possibly been due to the ability of quercetin and kaempferol to regulate multiple targets as suggested in silico. In current in vivo studies, the combined administration of quercetin and kaempferol showed an additive rather than a synergistic effect. This lack of synergy may be due to the same target for both compounds. However, the potential advantage of combining kaempferol with quercetin lies in its ability to enhance the bio-availability of kaempferol [31]. Moreover, the antidiabetic effect of kaempferol was significantly higher than standard drugs. Elevated levels of ALT and ALP can be due to hepatic cell damage leading to increased permeability and thereby bringing these enzymes into the blood [62]. However, in our study, no significant changes were observed in serum ALT, ALP, and bilirubin levels of experimental mice. Our current in vivo studies primarily focus on investigating these compounds in diabetes management, especially in glycemic control, insulin sensitivity, and in the control of dyslipidemia. Therefore, further studies of these compounds into diabetes-related complications such as cardiovascular complications, diabetic nephropathy, and neuropathy are required. The findings of our study suggest that these compounds could be potential multi-targeting antidiabetic drug candidates for diabetes management.

Diabetic individuals are more susceptible to various cancer types as compared to non-diabetics. It is also reported that certain antidiabetic agents, especially insulin glargine, promote cancer [50]. Therefore, developing drugs with both antidiabetic and anticancer activity is inevitable. In the current study, quercetin, kaempferol, and their combination significantly inhibited Huh-7 and HepG2 (cancer cell lines) proliferation. At the same time, no vivid cytotoxic effect was observed on the Vero cell line (normal cell line). The anticancer activity can be associated with the ability of both kaempferol and quercetin to cause cell cycle arrest at G1 and G2/M arrest and induce apoptosis in cancer cell lines. Quercetin has the potential to cause a pro-apoptotic effect. Quercetin and kaempferol inhibit cancer cell growth by down-regulating the expression of miR-21, PKC, COX-2, and PI3K/AKT/mTOR signaling, upregulating p53 and caspase 3, and enhancing the expression of IFN-α-regulated genes [31,63].

## 4. Materials and Methods

### 4.1. Reagents and Chemicals

Dulbecco’s Modified Eagle medium (cat. number 41965-047), nicotinamide (cat. number AC12827100), penicillin-streptomycin (Gibco^TM^, cat. number 115140-122), and fetal bovine serum (FBS) (Gibco^TM^, cat. number 10500-064) were obtained from Gibco (Thermo Fisher Scientific Inc., Waltham, MA, USA). Trypsin-EDTA (TLR01) was ordered from Caisson Labs (North Logan, UT, USA). Streptozotocin (cat. number sc-200719A) was purchased from Santa Cruz Biotechnology (Dallas, TX, USA). Ethanol (cat. number 459828) was purchased from Merck Co. (Darmstadt, Germany), and all other chemicals used during the experiment were purchased from Sigma-Aldrich (St. Louis, MO, USA). Dr. Nosheen Aslam, Associate Professor, GCUF, Pakistan, kindly provided us with quercetin and kaempferol.

### 4.2. Cell Lines

The HepG2, Huh-7 (hepatocellular carcinoma cell lines), and Vero (non-cancer cell line) were brought from the National Institute for Biotechnology and Genetic Engineering (NIBGE), Faisalabad, Pakistan, then sub-cultured and maintained at the Animal Cell Culture Lab (ACCL) as previously reported [64]. Upon reaching 70–80% confluency, cells were trypsinized and sub-cultured in 10 cm cell culture dishes. Dulbecco’s modified Eagle’s medium containing high glucose with 10% FBS, penicillin, and streptomycin (100 IU/mL and 100 μg/mL, respectively) was used. Cultured cell lines were maintained in a carbon dioxide incubator, maintaining a 37 °C temperature, 95% humidity, and 5% CO_2_.

### 4.3. In Silico Studies

#### 4.3.1. Drug Scan and ADMET Profiling

The druggability test was conducted using the “Lipinski rule of five” (http://www.scfbio-iitd.res.in/software/drugdesign/lipinski.jsp, accessed on 12 January 2022) [65]. The evaluation of ADMET-based properties of both compounds along with metformin was carried out using admetSAR (http://lmmd.ecust.edu.cn/admetsar2/, accessed on 12 January 2022) and Swiss ADME online analyzer (http://www.swissadme.ch/, accessed on 12 January 2022).

#### 4.3.2. Protein and Ligand Preparation

All receptor targets, namely CRP (PDB ID: 1gnh), DPP-IV (PDB ID: 1j2e), IL1 (PDB ID: 9ilb), PRARG (PDB ID: 1prg), PTP (PDB ID: 2nt7), and SLGT1 (PDB ID: 7sla), were retrieved from RCSB PDB (https://www.rcsb.org/structure, accessed on 30 January 2022) in PDB format. All the associated water and ligands with the 3D structures were removed using BIOVIA Discovery Studio 2021. Using MGL Tool 1.5.7, hydrogen and Kollman charges were added and saved in PDBQT format. The ligand structures were downloaded in SDF format from PubChem (https://pubchem.ncbi.nlm.nih.gov, accessed on 30 January 2022). The SDF format was converted to PDB using PyMol 2.5.4 and subsequently saved in PDBQT format [52].

#### 4.3.3. Docking Analysis

Docking analysis was carried out using AutoDock Vina 1.1.2 software. Grid parameters were calculated using auto dock 2.5.7. A *txt* file containing data related to the target, ligand, grid size, and geometry was saved. Docking analysis was carried out separately using the system command prompt, optimizing docking coordinates, and grid size. The PDBQT file was exported to PDB format for the output visualization, and binding modes were analyzed using BIOVIA Discovery Studio 2021 [52].

### 4.4. In Vitro Alpha-Amylase Inhibition Assay

The alpha-amylase enzyme inhibition assay was performed using the DNS method with minor modifications, as described in [45,66]. Forty microliters (40 µL) of the testing sample was added to 160 µL distilled water and 400 µL of 1% (*w*/*v*) starch solution. Subsequently, 200 µL of α-amylase (0.5 mg/mL) was added, and the reaction mixtures were incubated for 3 min at 25 °C. After incubation, 200 µL from the reaction tubes was transferred to other tubes containing 100 µL of dinitrosalicylic acid (DNS) reagent. The reaction was stopped by incubating it at 90 °C for 15 min. Finally, after adding 900 µL of distilled water, absorbance was measured at 540 nm using a double-beam spectrophotometer, A & E Labs; AE-S90-2D (Guangzhou, China). Similarly, the blank incubation included all reaction components except the testing sample, which was replaced by distilled water. Acarbose was used as positive control. The percentage inhibition of testing samples and acarbose was calculated using the formula below.

(1) $$\%\;Inhibition=\left(1-\frac{A_{unknown}}{A_{blank}}\right)\times 100$$

### 4.5. In Vivo Studies

#### 4.5.1. Experimental Animals

Male Balb/c mice used in this experiment, of average body weight (25–30 g), were obtained from Laboratory Animals and Research Centre (LARC), Akhuwat-FIRST Faisalabad, (Faisalabad, Pakistan), kept in transparent plastic cages, and provided with standard diet and an uninterrupted supply of clean and autoclaved water. A dark-and-light cycle of 12 h was maintained, and temperature and humidity were maintained around 22–25 °C and 50–60%, respectively. Ethical approval was issued by the Ethical Review Committee (Ref: AKT.FST/Misc./2022-31) and the Institutional Review Board (Ref: AKT.FST/Misc./2022-32) on ethical standards in animal experimentation. During experiments, all animals were treated humanely.

#### 4.5.2. Induction of Diabetes

After acclimatization, diabetes was induced in balb/c mice with slight modification, as described earlier [67]. All overnight-fasted mice were given a single dose of (i.p) injection of STZ 150 mg/kg body weight (BW), dissolved in citrate buffer of 0.1 m after 15 min of nicotinamide at 240 mg/kg BW (i.p). After 98 h of administration of STZ-NA, non-fasting hyperglycemia was confirmed. All mice groups were given respective doses of control, quercetin, kaempferol, their combination, and metformin for 28 days on alternate days. At the end of the experiment, the mice were euthanized, and serum samples were obtained by centrifuging at 3500 rpm for 10 min. Organs were also preserved by placing them in a 10% formaldehyde solution. Another batch of organs was stored at −80 °C for further analysis.

#### 4.5.3. Animal Grouping

Animals were grouped randomly. Group 1 received normal saline as control, group 2 received a single dose of STZ i.p + NA i.p (150 mg/kg B.W + 240 mg/kg B.W) served as diabetic control, group 3 containing STZ-NA diabetic mice received an oral dose of quercetin (20 mg/kg B.W), group 4 consisting of STZ-NA diabetic mice received an oral dose of kaempferol (5 mg/kg B.W), group 5 consisting of STZ-NA diabetic mice received the combination of quercetin and kaempferol (10 mg/Kg and (2.5 mg/Kg BW, respectively), and group 6 consisting of STZ-NA diabetic mice received an oral dose of metformin 50 mg/kg served as positive control.

#### 4.5.4. Blood Sampling and Glucose Level Analysis

During sacrifice and blood collection, a drop of blood around the neck was used to determine blood glucose level by using a glucometer (ON CALL Glucometer EZ II), and strips used were On-Call blood glucose strip LOT 395375, commonly used in clinical practices.

#### 4.5.5. Lipid Profile

Total cholesterol and triglycerides were determined using kits (Cholesterol Biomed Lot No. 150621 CHO-104400 and Triglyceride Biomed Lot No. 1701020 TG-1191000) respectively (Ebra, Miami, FL, USA).

#### 4.5.6. Levels of Bilirubin, ALT, and ALP

All diagnostic kits used were purchased from Ebra Mannheim (Ebra, Miami, FL, USA). Serum bilirubin levels were measured using kits (BILIRUBIN Ebra Lot No. 2002114 blt-0011). Serum ALT and ALP levels were determined to confirm liver damage, following the manufacturer’s protocols (ALT Ebra Lot No. BLT-00052 and ALK.PHOS Human Lot No. 0121 ETI-10700501).

#### 4.5.7. Total Antioxidant Capacity (TAC)

TAC capacity was estimated using the method described by Erel et al. [68]. This assay is based on the oxidation of ABTS (2,2′-azinobis (3-ethylbenzothiazoline-6-sulfonic acid) molecule to ABTS^+^ by hydrogen peroxide in an acidic medium to achieve a green color. The sample bleaches this color, and the bleaching rate is directly related to the amount of antioxidants in the sample. Briefly, 5 µL of the sample was added to 200 µL of reagent 1 (acetate buffer 0.4 mol/L pH 5.8). Further, 20 µL of reagent 2 (ABTS^+^ in acetate buffer 30 mmol/L at pH 3.6) was added and incubated at room temperature for 5 min. After incubation, absorbance was measured at 660 nm and the TAC of the samples was calibrated with Trolox. Blank absorbance was measured before mixing reagents 1 and 2. Final results are expressed as mmole of Trolox equivalent per liter.

### 4.6. Cell Viability Assay

An MTT (3-(4,5-dimethylthiazol-2-yl)-2,5-diphenyltetrazolium bromide) assay is a widely used colorimetric assay for assessing cell viability and proliferation. The assay was carried out to determine the effect of quercetin, kaempferol, and their combination on the viability of cancer and non-cancer cell lines. Cells were seeded on an ELISA plate at the concentration of 5 × 10^3^ per well. After 24 h of sub-culturing, cells were treated with quercetin (36 µg/mL), kaempferol (15 µg/mL) dissolved in 1% dimethyl sulfoxide (DMSO), their combination (quercetin 18 µg/mL + kaempferol 7.5 µg/mL), and doxorubicin (1.8 µg/mL) dissolved in water for 72 h. Each treatment was accessed in triplicates. Subsequently, the medium was removed, cells were washed with phosphate-buffered saline (PBS), and each cell was treated with MTT reagent (25 μL of 5 mg/mL MTT solution). After incubation for 4 h, the medium was removed, formazan crystals were dissolved in 125 μL of DMSO, and absorbance was taken at 570 nm using a microplate reader LTEK Co., Ltd. (Seongnam, Republic of Korea) [69].

### 4.7. Statistical Analysis

GraphPad Prism (GraphPad, 7.07) was used for data analysis. Data are expressed as mean ± SEM unless otherwise stated. All the differences between groups were determined by applying one-way ANOVA. * *p* < 0.05, ** *p* < 0.01, *** *p* < 0.001, and **** *p* < 0.0001 were considered statistically significant [70].

## 5. Conclusions

In light of the findings from this study, we conclude that quercetin and kaempferol act as multi-targeting antidiabetic agents, leading to glucose homeostasis as well as an improvement of lipid profiles and overall total antioxidant status. Both compounds showed their ability to inhibit and regulate multiple important targets in diabetes progression. In silico molecular docking revealed higher binding affinities for both compounds towards CRP, IL-1, DPP-IV, PPARG, PTP, and SGLT-1 compared to metformin, the commonly used antidiabetic drug. Moreover, both compounds possess desirable ADMET profiles with no toxic carcinogenic effects. These compounds also demonstrated good anticancer activity against HepG2 and Huh-7 cancer cell lines, showing no effect on the viability of Vero cell line (non-cancer). This study provides evidence to further investigate quercetin and kaempferol as potential multi-targeting antidiabetic drug candidates for diabetes management.

## Figures and Tables

**Table 1:** Drug-likeness parameters of quercetin, kaempferol, and metformin.

Compounds	Mol. WT (g/mol)	HBD	HBA	RBN	LogP	A	Violation
Quercetin	302.24	5	7	1	1.23	78.03	0
Kaempferol	286.24	4	6	1	1.58	76.01	0
Metformin	126.12	3	6	0	−1.38	37	0

**Table 2:** ADMET-related parameters of quercetin, kaempferol, and metformin.

	Quercetin	Kaempferol	Metformin
Absorption			
BBB	−	−	−
HIA	+	+	+
CaCo2 permeability	−	−	−
PGS	−	−	−
PGI	−	−	−
ROCT	−	−	−
Metabolism			
CYP3A4 Substrate	+	+	−
CYP2C9 Substrate	−	−	−
CYP2D6 Substrate	−	−	−
CYP3A4 Inhibition	+	+	−
CYP2C9 Inhibition	−	−	−
CYP2C19 Inhibition	−	+	−
CYP2D6 Inhibition	−	−	−
CYP1A2 Inhibition	+	+	−
Toxicity			
Ames toxicity	NAT	NAT	NAT
Carcinogens	Non-carcinogenic	Non-carcinogenic	Non-carcinogenic

BBB; blood–brain barrier, HIA; human intestinal absorption, PGS; P-glycoprotein substrate, PGI; P-glycoprotein inhibitor ROCT; renal organic cation transporter, NAT; non-Ames toxic. + and − represent presence and absence respectively.

**Table 3:** Predicted binding energies and detailed docking interaction of quercetin, kaempferol, and metformin with selected antidiabetic targets.

Target Receptor	Ligand		Binding H-Bond Residues	No. of H-Bonds
	Name	Binding Energy (Kcal/mol)		
CRP	Quercetin	−5.8	Thr126, Arg58, Lys57, Val127, Glu62, Gly128, Glu130, Pro29	5
	Kaempferol	−5.6	Arg58, Val127, Thr126, Lys57, Thr56, Gly128, Glu62, Pro29, Arg58	3
	Metformin	−4.2	Glu293, Gln145, Ser21, Ile134, Ser132 Gly143, Phe142	1
IL-1	Quercetin	−7.4	Leu62, Lys63, Glu64, Ser153, Asn7, Ser5, Ser43, Gly61, Lys65 Typ90, Pro91,Lys63	3
	Kaempferol	6.8	Leu62, Ala1, Arg4, Val3, Ser45, Ser,43 Gly61, Lys65, Asn66, Glu64, Ser5, Thre68	1
	Metformin	−5	Tyr68, Lu62, Lys65, Glu64, Lys63, Ser43, Pro91, Ser5, Ser54, Val486	1
DDPIV	Quercetin	−6.6	Thr516, Glu464, Lys 433, Tyr414, Ser484, Thr411, Ala465 Glu464, Val486	2
	Kaempferol	−6.5	Tyr416, Tyr414, Ser485, Lys433, Thr411, Ala465, Asp65, Lys566	1
	Metformin	−4.3	Ser485, Glu464, Lys433, Tyr416, Tyr414, Thr411, Ala465, Lys466, Val486	2
PPRG	Quercetin	−8.4	Ile326, Cys285, Glue291, Glu295, Pro227, Phe226, Met329, Lue333, Met334, Val339, Phe363, Tyr321, Ser289, Arg288, Leu330, Ala292	3
	Kaempferol	−8.3	Phe363, Ile326, Lue333, Met329, Phe226, Glu295, Glu291, Ser284, Tyr327, Arg288, Cys285, Leu330, Ala292	--
	Metformin	−5.3	Gln410, Asp441, Asp396, Val390, Met439, Gln437	3
PTP	Quercetin	8.2	Arg221, Lys120, Ser216, Asp181, Gln266, Gly220, Gln262, Gly183, Ile219, Val64, Asp48, Phe182, Tyr46, Ala217	2
	Kaempferol	−8.3	Arg221, Val49, Ile219, Ser216, Asp181, Gly183, Gln226, Gly220, Gln261, Asp48	2
	Metformin	4.8	Ser80, Leu204, Gly209, Ser205, His208, Val221, pro210, Ser203, Arg79, Pro206, Gln78	6
SGLT-1	Quercetin	−8.4	Asp161, Tyr290, Ser81, Val160, Ile456, Thr156, Phe101, Gln457, Leu286, Trp289, Ala287, Ser72, Ile397, Ser393	2
	Kaempferol	−8.4	Asp161, Ser71, Tyr290, Ser81, Val160, Ile456, Thr156, Phe101, Ser393, Asn78, Trp289, Gln457, Leu286, Ser460	3
	Metformin	−5.3	Ser77, Ser81, Ser393, Val160, Lys157, Ile456, Thr156, Gly82, Asn78, Ile79	2

CRP (PDB ID: 1gnh), DPP-IV (PDB ID: 1j2e), IL1 (PDB ID: 9ilb), PRARG (PDB ID: 1prg), PTP (PDB ID: 2nt7), and SLGT1 (PDB ID: 7sla). Quercetin (PubChem CID: 5280343), Kaempferol (PubChem CID: 5280863) and Metformin (PubChem CID: 4091).

**Table 4:** Effect of quercetin, kaempferol, and their combination on level of serum ALT, ALP, and bilirubin.

Treatments	ALT (IU/L)	ALP (IU/L)	Bilirubin (mg/dL)
Control	69.0 ± 11	87.3 ± 6.3	0.55 ± 0.03
STZ-NA + Veh	80.5 ± 17	89 ± 07	0.64 ± 0.04
STZ-NA + Quer	68.0 ± 07	79.5 ± 2	0.605 ± 0.02
STZ-NA + Kaem	78.5 ± 08	78.5 ± 12	0.565 ± 0.11
STZ-NA + Quer + Kaem	85 ± 6.8	93 ± 15	0.625 ± 0.01
STZ-NA + Met	80.5 ± 18.9	92 ± 14	0.47 ± 0.03

Values are expressed as means ± SEM, where *n* = 3. Statistical analysis was applied to find level of significance as in Figure 4. Ctrl, normal control; Veh, vehicle (diabetic control); Quer, quercetin; Kaem, kaempferol; and Met, metformin (positive control).

## Data Availability

All the data obtained from experiments are included in the manuscript.
